# The Value of Working-Men's Support

**Published:** 1912-02-24

**Authors:** T. Morgan

**Affiliations:** Secretary Pontypool and District Hospital.


*To the Editor of* The Hospital.

### THE VALUE OF WORKING-MEN'S SUPPORT.

*To the Editor of* The Hospital.

Sir,—On several occasions you have received reports that, in consequence of the Insurance Act, some hospital secretaries have had to record a falling-off in contributions to the funds of their institutions. I am pleased to report as follows for the month of January :—

Receipts, January 1911, £322 3s.; receipts, January 1912, £392 14s. 3d.; an increase of £70 11s. 3d. for the first month of the year, as against the corresponding period of last year.—Faithfully yours,

February 16. T. Morgan.
Secretary Pontypool and District Hospital.

[The above letter confirms our statement in The Hospital of November 11, 1911, that "this is eminently a working-man's hospital, and the financial year, we understand, is showing that Mr. Morgan is well maintaining the hospital's grip on their regard." We hope this may not prove an exceptional case.—Ed. The Hospital.]

[Owing to pressure of space, we have had to hold over a letter from Dr. J. C. McWalter on "A State Nursing Service."]

